# Mortality Among Pediatric Patients With Acute Lymphoblastic Leukemia in Sweden From 1988 to 2017

**DOI:** 10.1001/jamanetworkopen.2022.43857

**Published:** 2022-11-28

**Authors:** Thomas Björk-Eriksson, Martina Boström, Ing-Liss Bryngelsson, Päivi M. Lähteenmäki, Marianne Jarfelt, Marie Kalm, Daniel S. Olsson

**Affiliations:** 1 Department of Oncology, Institute of Clinical Sciences, Sahlgrenska Academy, University of Gothenburg, Gothenburg, Sweden; 2 Regional Cancer Centre West, Western Sweden Healthcare Region, Gothenburg, Sweden; 3 Medical Affairs, Neurology, Go North Medical AB, Gothenburg, Sweden; 4 Department of Pharmacology, Institute of Neuroscience and Physiology, Sahlgrenska Academy, University of Gothenburg, Gothenburg, Sweden; 5 Department of Occupational and Environmental Medicine, Faculty of Medicine and Health, Örebro University, Örebro, Sweden; 6 Swedish Childhood Cancer Registry, Karolinska Institute, Stockholm, Sweden; 7 Department of Oncology at Sahlgrenska University Hospital, Gothenburg, Sweden; 8 Cardiovascular, Renal and Metabolism, BioPharmaceuticals R&D, AstraZeneca, Gothenburg, Sweden; 9 Department of Internal Medicine and Clinical Nutrition, Institute of Medicine, Sahlgrenska Academy, University of Gothenburg, Gothenburg, Sweden; 10 Department of Endocrinology at Sahlgrenska University Hospital, Gothenburg, Sweden

## Abstract

**Importance:**

Acute lymphoblastic leukemia (ALL) constitutes 20% to 30% of all pediatric cancers. The 5-year overall survival among pediatric patients with ALL in high-income countries such as Sweden is currently more than 90%, but long-term unselected nationwide mortality data and mortality data in relation to the general population are lacking.

**Objective:**

To compare mortality between pediatric patients with ALL and the general population during a 30-year period in Sweden and to assess the incidence of ALL in Sweden.

**Design, Setting, and Participants:**

This cohort study included pediatric patients (aged <18 years) with a morphologically verified ALL diagnosis in the Swedish Cancer Register and/or at least 2 ALL diagnoses in the Swedish National Patient Register between January 1, 1988, and December 31, 2017. Data were cross-linked to the Swedish Cause of Death Register. Data were analyzed from May 2019 to January 2022.

**Main Outcomes and Measures:**

The main outcomes were mortality among patients with ALL compared with that in the general population and mortality in different subgroups within the cohort. Standardized mortality ratios (SMRs) were calculated using the general Swedish population as a reference. Within-cohort survival analyses were performed.

**Results:**

A total of 2397 patients (1354 [56%] male; mean [SD] age at diagnosis, 6.1 [4.7] years) were included in the study. The mean (SD) incidence of pediatric ALL during the study period was 4.11 (0.60) cases per 100 000 persons per year (females, 3.68 [0.65] cases per 100 000 persons per year; males, 4.52 [0.81] cases per 100 000 persons per year; *P* < .001). The observed number of deaths among pediatric patients with ALL was 409 vs the 9.5 deaths expected in the general population, resulting in an overall SMR of 43.1 (95% CI, 39.0-47.5); females had a higher SMR than males (57.8 [95% CI, 49.5-67.2] vs 34.5 [95% CI, 32.0-41.4]; *P* < .001). Analysis within the cohort showed a continued decrease in survival throughout the 30-year follow-up. The association between calendar year of ALL diagnosis, corresponding with different ALL treatment protocols, and mortality showed the lowest survival for the 1988-1991 group and the highest for the 2008-2017 group (χ^2^ = 20.3; *P* < .001).

**Conclusions and Relevance:**

In this cohort study, a consistently high SMR was seen among pediatric patients with ALL. Within the ALL cohort, survival evolved to a similar extent as in the young general population of Sweden. Furthermore, survival among patients with ALL decreased throughout the whole follow-up period without any trend difference after the 5-year follow-up time point. The changes in ALL treatment protocols were associated with overall improved absolute survival over time.

## Key Points

**Question:**

Has mortality among pediatric patients with acute lymphoblastic leukemia (ALL) in Sweden improved compared with the general population?

**Findings:**

In this cohort study of 2397 Swedish pediatric patients with ALL, the number of deaths from 1988 to 2017 was substantially higher than the expected number in the general population, resulting in an overall high standardized mortality ratio (SMR). Females had a higher SMR than males.

**Meaning:**

In this study, survival in Swedish pediatric patients with ALL evolved to a similar extent as in the young general population, leading to a consistently high SMR during the study period.

## Teaser

This cohort study compares mortality among Swedish pediatric patients diagnosed with acute lymphoblastic leukemia with that in the general population of Sweden from 1988 to 2017.

## Introduction

Acute lymphoblastic leukemia (ALL) is the most common childhood cancer, accounting for approximately 20% to 30% of pediatric cancers.^1^ In Sweden, the respective figure is 24% for children younger than 15 years at diagnosis.^2^ The prognosis of pediatric ALL has improved substantially over the past 50 years in high-income countries such as Sweden, from 5-year overall survival of approximately 5% in the 1970s to more than 90% today.^3,4^ Survival improvement started with the introduction of prophylactic cranial irradiation.^5^ Later, enhancements in risk stratification, consequent treatment intensity adaptations, and treatment of acute adverse effects played a central role in further survival improvement.^3,4,6^

However, the first step of increased survival was accompanied by irradiation-induced neurocognitive and pituitary deficiencies.^7,8^ Consequently, irradiation was gradually replaced in most cases by systemic high-dose methotrexate to prevent central nervous system (CNS) relapse.^9^ In Sweden, collaboration between pediatric clinics at university hospitals resulted in national ALL treatment protocols from 1968 until 1988. Thereafter, Sweden joined the Nordic Organization of Hematology and Oncology (NOPHO) ALL trials (NOPHO ALL1992,^10^ ALL2000,^11^ and ALL2008^12^), thus delineating 4 major phases in the development of pediatric ALL treatment. The 1992 treatment protocol was the first to avoid CNS irradiation except for individuals with very high-risk disease or CNS involvement at diagnosis.^11^

Despite improved survival rates, cancer remains one of the most common causes of death in children. To our knowledge, there have been no population-based studies of excess mortality from ALL diagnosis in large numbers of pediatric patients. While many studies have specifically investigated late mortality in large cohorts of 5-year survivors of pediatric ALL in the US,^13,14,15^ Britain,^16^ and Nordic countries,^17^ to our knowledge, none have studied excess mortality from diagnosis. The ability to interlink data between different Swedish health registries permits the study of nationwide patient cohorts, avoiding selection bias over extensive periods. The aim of our study was to investigate mortality among pediatric patients with ALL compared with that in the nationwide Swedish population over an extensive 30-year period and to analyze the incidence of pediatric ALL in Sweden over this period.

## Methods

### Study Design

The unique Swedish personal identification number identifies individuals across national registries, creating unique conditions for register-based medical research.^18^ Our study was conducted as a national registry-based cohort study of pediatric patients with ALL. Patients were identified in the Swedish National Patient Register (Patient Register), which contains information on every patient visit within the Swedish hospital system since 1987, or from the Swedish Cancer Register (Cancer Register). The Cancer Register is a morphologic registry of very high quality, in which it has been mandatory since 1958 for all health care practitioners to report newly detected malignant tumors, including morphologic and laboratory data, as well as cases diagnosed at autopsy. It has very high coverage (>96%) of the Swedish population.^19,20^ Information available in the Cancer Register is of 3 different types: data on the patient, medical data, and follow-up data. The Swedish Cause of Death Register has recorded the time and cause of all deaths in Sweden since 1952.^21^ In the current study, the personal identification number was used to merge data from the 3 registers. The National Board of Health and Welfare secures the high quality of the national health registries.^22,23,24^ The study was approved by the Regional Ethical Review Board in Gothenburg, Sweden, and by the National Board of Health and Welfare, Sweden. A waiver of informed consent was granted by the Regional Ethical Review Board in Gothenburg, Sweden, and by the National Board of Health and Welfare, Sweden, because the data were deidentified. The design and method of the study as well as the article followed, in all relevant parts, the Strengthening the Reporting of Observational Studies in Epidemiology (STROBE) reporting guideline.

### Study Population

The study population included all pediatric patients (<18 years of age at diagnosis) diagnosed with ALL in Sweden between January 1, 1988, and December 31, 2017. To ensure high accuracy in the patient inclusion process, a combination of 2 registers was used together with a washout period. All patients with a morphologically verified ALL diagnosis in the Cancer Register were included in the cohort. In addition, the cohort included all patients with at least 2 ALL diagnoses from inpatient visits in the Patient Register. A washout period between January 1, 1987, and December 31, 1987, was used to ensure that only newly diagnosed patients were included from the Patient Register. Specific inclusion criteria used in the Cancer Register (including morphologic codes) and the Patient Register are presented in eTable 1 in the Supplement.

### Data Retrieval for the Patient Cohort

The follow-up period, during which patients were studied regarding mortality, was from inclusion into the study until death or the end of the study (December 31, 2017). Date of birth, sex, and year of ALL diagnosis were collected from the Patient Register and the Cancer Register. Causes of death were obtained from the Swedish Cause of Death Register and classified in accordance with the *International Statistical Classification of Diseases and Related Health Problems, Tenth Revision* (*ICD-10*) (or translated from the *International Classification of Diseases, Ninth Revision* [*ICD-9*]); furthermore, cause-specific mortality was studied for all malignant tumors; malignant neoplasms of the brain; malignant neoplasms of lymphoid, hematopoietic, and related tissues; ischemic heart disease; and cerebrovascular disease (eTable 2 in the Supplement).

### Statistical Analysis

Data were analyzed from May 2019 to January 2022. Continuous variables were presented as mean (SD) and categorical variables as number (percentage). The Student *t* test was used for comparison of age at diagnosis between sexes. A 1-sample binomial test was performed to study sex distribution. Kaplan-Meier survival plots and log-rank tests were used to make internal comparisons of mortality between nonoverlapping subgroups.

Person-years at risk were calculated from study inclusion (diagnosis) until death or the end of the study and stratified according to sex, 5-year age groups, and 1-year calendar periods. The expected number of cases for each stratum was calculated using the general Swedish population for every calendar year and 5-year age group as reference. The observed number of deaths among patients with ALL was compared with the expected number in the general population, and standardized mortality ratios (SMRs) were calculated. The 95% CIs were calculated assuming a Poisson distribution of the observed numbers. The SMRs for nonoverlapping subgroups were compared.^25^ In the subanalysis in which the patients were grouped depending on calendar year of diagnosis, all patients had 4 years of follow-up if they had not died during that time. A sensitivity analysis (SMR) was performed regarding the year of ALL diagnosis. For each of the 4 subgroups, the reference data started in 1988 instead of the year of diagnosis, thereby indirectly evaluating the effect of increased life expectancy in the general population. All statistical analyses were carried out using Stata, version 15 (StataCorp LLC), and IBM SPSS, version 25 (IBM Corp). Significance was set at 2-sided *P* < .05.

## Results

### Patient Characteristics

A total of 2397 patients (1043 [44%] female; 1354 [56%] male) with pediatric ALL, with a mean (SD) age at diagnosis of 6.1 (4.7) years, were included in the study (Table 1). Most of the patients (2084 [87%]) were included from the Cancer Register.

**Table 1.** Baseline Demographic and Clinical Characteristics of the Cohort of Pediatric Patients With ALL

Characteristic	Patients (N = 2397)^a^	*P* value
Sex		
Female	1043 (44)	<.001
Male	1354 (56)	
Age at ALL diagnosis, mean (SD), y		
All	6.1 (4.7)	<.001
Female	5.8 (4.7)	
Male	6.4 (4.7)	
Age group at ALL diagnosis, y		
0-4	1182 (49)	NA
5-10	621 (26)	
11-15	409 (17)	
16-17	185 (8)	
Calendar year at ALL diagnosis		
1988-1991	296 (12)	NA
1992-1999	646 (27)	
2000-2007	640 (27)	
2008-2013	499 (21)	
Follow-up duration, mean (SD), y	12.4 (9.0)	NA
Inclusion from register		
Swedish Cancer Register and Swedish National Patient Register^b^	2055 (86)	NA
Swedish Cancer Register alone	29 (1)	
Swedish National Patient Register alone^b^	313 (13)	
Deaths	409 (17)	NA

Abbreviations: ALL, acute lymphoblastic leukemia; NA, not applicable.

^a^ Data are presented as the number (percentage) of patients unless otherwise indicated.

^b^ Two inpatient visits fulfilling the inclusion criteria were needed to be included from the Swedish Patient Register.

**Figure 1.**
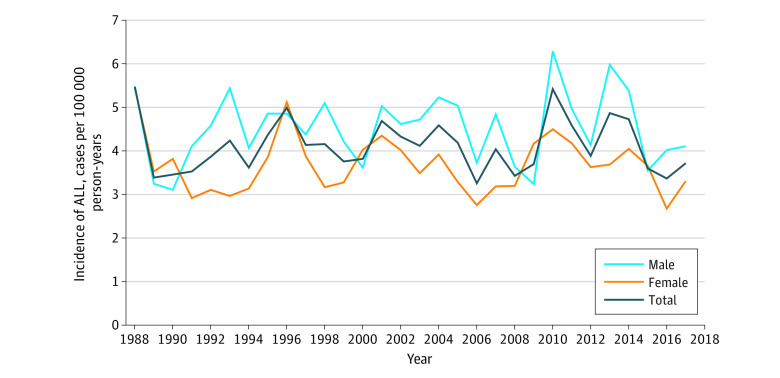
Incidence of Pediatric Acute Lymphoblastic Leukemia (ALL) in Sweden From 1988 to 2017 The incidence was calculated using the mean Swedish annual general population.

The mean (SD) incidence of pediatric ALL during the whole study period (1988-2017) was 4.11 (0.60) cases per 100 000 persons per year (Figure 1), with males having a higher incidence than females (mean [SE], 4.52 [0.81] vs 3.68 [0.65] cases per 100 000 persons per year; *P* < .001). The mean (SD) follow-up period was 12.4 (9.0) years (range, 0-30 years) (Table 1). Furthermore, patients were divided into 4 groups depending on age at diagnosis (0-4, 5-10, 11-15, and 16-17 years), with the youngest group being the largest (1182 [49%]). Patients were also assigned to 4 groups according to different calendar periods depending on the date of ALL diagnosis to match the use of different national treatment protocols (Table 1).

### Overall Mortality Compared With the General Population

The observed number of deaths among pediatric patients with ALL was 409 vs the 9.5 expected in the general population, resulting in an overall SMR of 43.1 (95% CI, 39.0-47.5) (Table 2). Females had a higher SMR than males (57.8 [95% CI, 49.5-67.2] vs 34.5 [95% CI, 32.0-41.4]; *P* < .001). Pediatric patients diagnosed between 0 and 4 years of age had lower mortality (SMR, 35.0; 95% CI, 29.7-41.0) compared with patients diagnosed at an older age (5-10 years: SMR, 46.9; 95% CI, 37.9-57.4; *P* = .03; 11-15 years: SMR, 52.3; 95% CI, 42.8-63.4; *P* = .002; and 16-17 years: SMR, 52.2; 95% CI, 39.4-67.7; *P* = .01) (Table 2). The SMR was similar for the different subgroups depending on the calendar year of diagnosis (Table 2). On the contrary, the sensitivity analysis using historical reference data showed decreasing SMRs with time. The SMR for 5-year survivors (baseline: 5 years after diagnosis) was 12.3 (95% CI, 9.8-15.3) (observed deaths, 81; expected, 6.6) in the whole group (n = 1683) and 9.0 (95% CI, 6.5-12.1) and 21.3 (95% CI, 15.1-29.2) for males and females, respectively.

**Table 2.** Standardized Mortality Ratios for Pediatric ALL in Sweden

Characteristic	Deaths among pediatric patients with ALL, No.	Expected deaths in the general population, No.	Standardized mortality ratio (95% CI)
Overall mortality			
All	409	9.5	43.1 (39.0-47.5)
Females	171	3.0	57.8 (49.5-67.2)
Males	238	6.5	34.5 (32.0-41.4)
Age at ALL diagnosis, y			
0-4	154	4.4	35.0 (29.7-41.0)
5-10	94	2.0	46.9 (37.9-57.4)
11-15	105	2.0	52.3 (42.8-63.4)
16-17	56	1.1	52.2 (39.4-67.7)
Date at ALL diagnosis^a^			
1988-1991	56	0.5	114.8 (86.7-149.1)
1992-1999	91	0.8	111.8 (90.0-137.3)
2000-2007	90	0.7	132.6 (106.6-163.0)
2008-2013	71	0.7	109.3 (85.3-137.8)
Cause-specific mortality, *ICD-10* chapter^b^			
1	2	0.1	15.0 (1.8-54.2)
2			
All	382	1.2	327.6 (295.5-362.1)
Females	161	0.5	334.7 (285.0-390.6)
Males	221	0.7	322.5 (281.4-368.0)
3	3	0.1	55.4 (11.4-161.8)
9	2	0.5	4.4 (0.5-16.0)
10	3	0.2	19.3 (4.0-56.3)
17	3	0.8	4.0 (0.8-11.6)
18	7	0.5	13.4 (5.4-27.7)
20	5	4.5	1.1 (0.4-2.6)
Specific cause of mortality			
Hematologic malignant tumors			
All	356	0.2	1551.1 (1394.1-1720.9)
Females	150	0.1	1878.4 (1589.9-2204.3)
Males	206	0.2	1376.4 (1198.5-1577.8)
Brain tumors	4	1.1	15.4 (4.2-39.3)

Abbreviations: ALL, acute lymphoblastic leukemia; *ICD-10*, *International Statistical Classification of Diseases and Related Health Problems, Tenth Revision*.

^a^ All patients had 4 years of follow-up except those who died during the study period.

^b^ *ICD-10* chapters: 1, certain infectious and parasitic diseases; 2, neoplasms; 3, diseases of the blood and blood-forming organs and certain disorders involving the immune mechanism; 9, diseases of the circulatory system; 10, diseases of the digestive system; 17, congenital malformations, deformations, and chromosomal abnormalities; 18, symptoms, signs, and abnormal clinical and laboratory findings, not elsewhere classified; and 20, external causes of morbidity.

Overall, 5-, 10-, and 15-year survival rates for the entire cohort were 85.5%, 82.6%, and 81.7%, respectively. Corresponding 5-, 10-, and 15-year survival rates were 84.7%, 82.0%, and 81.0%, respectively, among males, and 86.5%, 83.3%, and 82.5%, respectively, among females. When analyzing the outcomes among the 5-year survivors, their long-term survival rates were 96.4% and 94.6% at 5- and 10-year follow-up, respectively; corresponding survival rates were 96.5% and 94.9% among males and 96.2% and 94.2% among females at 5- and 10-year follow-up, respectively.

### Cause-Specific Mortality

When analyzing mortality from causes within specific *ICD-10* chapters, there were no obvious increases except for causes in *ICD-10* chapter 2, which includes the codes for hematologic malignant tumors (Table 2). Within the subgroup of deaths due to hematologic malignant tumors (356 events), 88% (313 events) and 10% (35 events) had specific ALL and acute myeloblastic leukemia *ICD-10* or *ICD-9* codes as their cause of death, respectively. However, when investigating cause-specific mortality, the SMR for brain tumors was increased (4 events; SMR, 15.4; 95% CI, 4.2-39.3). These 4 patients died from (*ICD-10* or *ICD-9* code) malignant neoplasm of frontal lobe (C71.1) (n = 1), malignant neoplasm of cerebellum (C71.6) (n = 1), and malignant neoplasms of brain, unspecified location (C71.9 and C191.9) (n = 2).

### Internal Analysis of Overall Mortality

**Figure 2.**
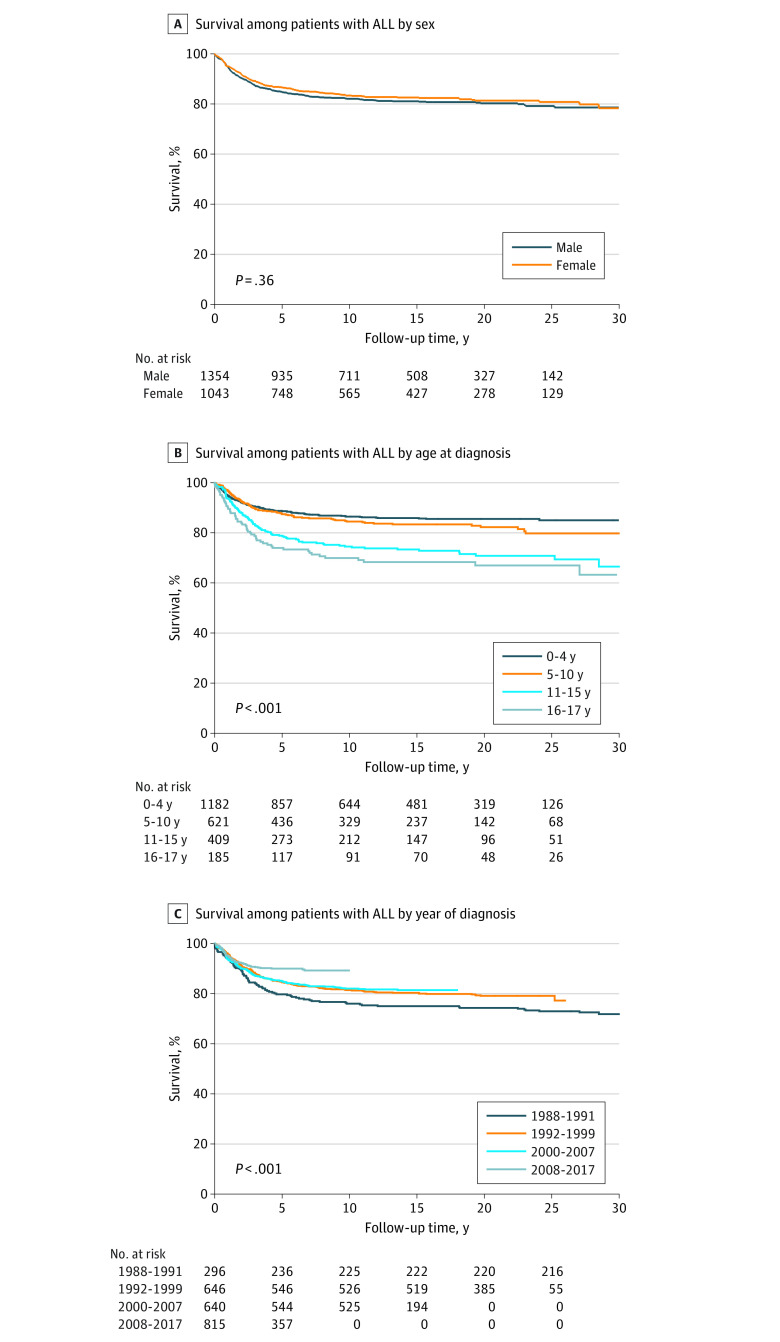
Kaplan-Meier Survival Curves for Pediatric Acute Lymphoblastic Leukemia (ALL) in Sweden From 1988 to 2017 The log-rank test was used to test for significant differences between the groups.

The Kaplan-Meier survival curve showed that the largest decrease in survival was seen within the first 5 years after the ALL diagnosis, although survival continued to decrease throughout the whole 30-year follow-up period (Figure 2A). Furthermore, there was no significant difference in overall survival between males and females with ALL (log-rank test, χ^2^ = 0.85; *P* = .36) (Figure 2A). Survival was lower with older age at ALL diagnosis when analyzing the 4 different age groups at diagnosis (log-rank test, χ^2^ = 56.5; *P* < .001) (Figure 2B). When analyzing the association between the calendar year of ALL diagnosis (which corresponded with different ALL treatment protocols) and survival, the lowest survival was seen for the 1988-1991 group and the highest for the 2008-2017 group (log-rank test, χ^2^ = 20.3; *P* < .001) (Figure 2C). In addition, patients diagnosed during 1992 or later had better survival than patients diagnosed before 1992 (log-rank test, χ^2^ = 9.99; *P* = .002). No significant difference in survival was found between patients diagnosed from 1992 to 1999 and those diagnosed from 2000 to 2007 (log-rank test, χ^2^ = 0.23; *P* = .63). However, significantly better survival was found in the latest group, diagnosed between 2008 and 2017, compared with patients diagnosed between 2000 and 2007 (log-rank test, χ^2^ = 8.65; *P* = .003).

## Discussion

To our knowledge, this is the first large population-based study to investigate all-cause mortality among pediatric patients with ALL (<18 years of age at diagnosis) with that in the general population as reference and with an extensive follow-up duration. The large number of patients included in the study and the up to 30-year follow-up period enabled a comparison of outcomes for the different treatment protocols used over time. The main findings are that survival continued to decrease throughout the whole follow-up period beyond the first 5 years after diagnosis and that improvement was seen with updated treatment protocols. Of interest, females had a higher SMR than males, and excess mortality was associated with age group at diagnosis.

The SMRs for the 4 different treatment protocol periods overlapped, showing no relative survival improvement during the study period. However, when performing the internal comparison in the study cohort between the different protocol periods, we observed an increase in absolute survival with more recent treatment protocol periods. For example, the current study results support earlier findings that prophylactic cranial irradiation (performed in patients diagnosed before 1992) can be safely omitted and replaced by high-dose methotrexate in the treatment of childhood ALL if an effective risk-adjusted chemotherapy is used (performed in patients diagnosed after 1992).^11,26^ Also, in the NOPHO ALL2008 protocol, treatment was intensified in high-risk patients, and the use of asparaginase and high-dose methotrexate with coadministration of mercaptopurine was increased in all risk groups; both of these protocol changes contributed to a tolerable and effective protocol.^12^ Internal analysis in our current study also showed better survival among patients diagnosed in 2008 or later compared with those with an earlier diagnosis. This discrepancy between the external comparison with the general population and the internal comparison may be explained by the improved overall survival in the Swedish pediatric population. The mortality rate among Swedish children younger than 18 years decreased from 31 to 23 deaths per 100 000 children per year from 2000 to 2014, which represents an overall decrease of 26%.^27^ Furthermore, the mortality rate among Swedish children younger than 5 years is among the lowest in the world.^28^ This may in part explain why pediatric cancer has been the most common cause of death among Swedish children and adolescents since 2006 despite the historically high survival rates among patients with pediatric cancer, including ALL, in Sweden. Even though survival among patients with ALL has improved significantly over time, resulting in decreased mortality, the same level of improved survival has occurred in the general population, resulting in an unchanged SMR across the ALL protocols.^6^ This interpretation was further strengthened by a sensitivity analysis performed with historical reference data. Similar improvements to those in Sweden have also been demonstrated in European children with all types of leukemia, with an annual reduction of 4% in mortality.^29^

Survival among patients with ALL in Sweden has, as in most countries, increased during the past decades. However, in comparison with the general population, mortality among pediatric patients with ALL has been high. In our study, we demonstrated 40 times higher mortality among pediatric patients with ALL than in the general population (SMR, 43.1). The mortality rate was highest during the first 5 years after diagnosis. This might partly be explained by the risk for death from treatment-related toxic effects and relapse. However, the deaths caused by toxic effects during ALL treatment cannot be separated from those caused by leukemia in the registries. The causes of death in this study were mainly due to hematologic diseases, which is in line with a Nordic study in which the relapse incidence was 19% from 1992 to 2011.^30^ It is also notable that survival continued to decrease in the period after the 5-year follow-up, hence showing the importance of supporting survivors over a considerable period.

The incidence of ALL has been previously reported to be higher among males than among females,^31,32^ which was also found in this study. It has also been reported that females treated for childhood ALL have a higher survival rate than males.^31,32,33^ This was also seen in earlier Nordic analyses.^34,35^ However, we found no significant difference in overall mortality between males and females (internal analysis) in our study. Similar mortality between the sexes was also reported in the NOPHO ALL2000 trial.^11^ On the contrary, when comparing female patients who have ALL with the general female population and male patients who have ALL with the general male population, we found a considerably higher SMR for females than for males, which might partly be explained by the relatively lower mortality among females in the general population. In addition, in the NOPHO ALL1992 and ALL2000 trials, females were twice as likely to experience treatment-related mortality than were males.^11^ In a report from the Children’s Oncology Group,^36^ it was stated that high-risk female patients with ALL experienced significantly more treatment-related deaths than did males. Furthermore, a study from the UK showed that females had significantly more infection-related deaths during ALL induction therapy.^37^ On the contrary, in a Nordic study, a higher risk for death due to relapse was seen in boys compared with girls, but the study did not find any difference in treatment-related causes of death between sexes.^28,38^ The underlying biological mechanisms for these sex differences are still poorly understood and warrant further investigation.

The internal analysis of overall mortality in our study also showed that survival was lower with older age at ALL diagnosis when analyzing the 4 different age groups at diagnosis. This finding of a higher mortality rate among adolescent patients with ALL has been previously described.^31,39^ A higher mortality rate was shown to be associated with a higher frequency of T-cell ALL and higher postinduction minimal residual disease leading to the need for high-risk therapy, which is associated with a more frequent need for stem cell transplants.^12^ Also, using other age bands (1-4, 5-9, 10-14, and 15-19 years) showed similar mortality trends.^31^ That study, however, also specifically analyzed survival among children younger than 1 year, who had the highest mortality.^31^ In our study, instead, we selected to use 4 age bands (0-4, 5-10, 11-15, and 16-17 years), hence the slightly different results.

### Strengths and Limitations

A strength of our study is the use of reliable national population-based registries in Sweden spanning an extensive 30-year period. By interlinking data from several Swedish health registries, we were able to compare both early and late mortality rates among pediatric patients with ALL with those in the general population in an unbiased manner over a long period.

This study also has limitations. One weakness is that the registries used in this study did not provide any detailed information about patients, conditions such as Down syndrome, immunophenotype, unfavorable cytogenetics, treatment, or relapse, which prevented us from conducting any further analyses of our findings.

## Conclusions

In this cohort study, a consistently high SMR was seen among pediatric patients with ALL in Sweden. Within the ALL cohort, survival evolved to a similar extent as in the young general population of Sweden. Of interest, survival among patients with ALL decreased throughout the whole follow-up period. The changes in ALL treatment protocols over time were associated with overall improved absolute survival. We hope that new treatment strategies for pediatric patients with ALL will result in a reduction of mortality compared with the general population.

## Notes

Supplement. **eTable 1.** Inclusion Criteria Used in the Cancer Register and the Patient Register **eTable 2.** Codes for Cause-specific Mortality

## References

[1] Siegel DA, Henley SJ, Li J, Pollack LA, Van Dyne EA, White A. Rates and trends of pediatric acute lymphoblastic leukemia—United States, 2001-2014. MMWR Morb Mortal Wkly Rep. 2017;66(36):950-954. doi:10.15585/mmwr.mm6636a3 28910269 PMC5657918

[2] Årsrapport 2020 Svenska Barncancerregistret. Accessed August 31, 2021. https://cceg.ki.se/documents/Arsrapport_SBCR_2020.pdf

[3] Hunger SP, Mullighan CG. Acute lymphoblastic leukemia in children. N Engl J Med. 2015;373(16):1541-1552. doi:10.1056/NEJMra1400972 26465987

[4] Malard F, Mohty M. Acute lymphoblastic leukaemia. Lancet. 2020;395(10230):1146-1162. doi:10.1016/S0140-6736(19)33018-1 32247396

[5] Pinkel D, Simone J, Hustu HO, Aur RJ. Nine years’ experience with “total therapy” of childhood acute lymphocytic leukemia. Pediatrics. 1972;50(2):246-251. doi:10.1542/peds.50.2.246 4505343

[6] Inaba H, Mullighan CG. Pediatric acute lymphoblastic leukemia. Haematologica. 2020;105(11):2524-2539. doi:10.3324/haematol.2020.247031 33054110 PMC7604619

[7] Gurney JG, Ness KK, Sibley SD, . Metabolic syndrome and growth hormone deficiency in adult survivors of childhood acute lymphoblastic leukemia. Cancer. 2006;107(6):1303-1312. doi:10.1002/cncr.22120 16894525

[8] Goldsby RE, Liu Q, Nathan PC, . Late-occurring neurologic sequelae in adult survivors of childhood acute lymphoblastic leukemia: a report from the Childhood Cancer Survivor Study. J Clin Oncol. 2010;28(2):324-331. doi:10.1200/JCO.2009.22.5060 19917844 PMC2815720

[9] Moe PJ, Seip M, Finne PH. Intermediate dose methotrexate (IDM) in childhood acute lymphocytic leukemia in Norway. Preliminary results of a national treatment program. Acta Paediatr Scand. 1981;70(1):73-79. doi:10.1111/j.1651-2227.1981.tb07176.x 6971042

[10] Schmiegelow K, Björk O, Glomstein A, . Intensification of mercaptopurine/methotrexate maintenance chemotherapy may increase the risk of relapse for some children with acute lymphoblastic leukemia. J Clin Oncol. 2003;21(7):1332-1339. doi:10.1200/JCO.2003.04.039 12663723

[11] Schmiegelow K, Forestier E, Hellebostad M, ; Nordic Society of Paediatric Haematology and Oncology. Long-term results of NOPHO ALL-92 and ALL-2000 studies of childhood acute lymphoblastic leukemia. Leukemia. 2010;24(2):345-354. doi:10.1038/leu.2009.251 20010622

[12] Toft N, Birgens H, Abrahamsson J, . Results of NOPHO ALL2008 treatment for patients aged 1-45 years with acute lymphoblastic leukemia. Leukemia. 2018;32(3):606-615. doi:10.1038/leu.2017.265 28819280

[13] Armstrong GT, Liu Q, Yasui Y, . Late mortality among 5-year survivors of childhood cancer: a summary from the Childhood Cancer Survivor Study. J Clin Oncol. 2009;27(14):2328-2338. doi:10.1200/JCO.2008.21.1425 19332714 PMC2677921

[14] Essig S, Li Q, Chen Y, . Risk of late effects of treatment in children newly diagnosed with standard-risk acute lymphoblastic leukaemia: a report from the Childhood Cancer Survivor Study cohort. Lancet Oncol. 2014;15(8):841-851. doi:10.1016/S1470-2045(14)70265-7 24954778 PMC4142216

[15] Armstrong GT, Chen Y, Yasui Y, . Reduction in late mortality among 5-year survivors of childhood cancer. N Engl J Med. 2016;374(9):833-842. doi:10.1056/NEJMoa1510795 26761625 PMC4786452

[16] Reulen RC, Winter DL, Frobisher C, ; British Childhood Cancer Survivor Study Steering Group. Long-term cause-specific mortality among survivors of childhood cancer. JAMA. 2010;304(2):172-179. doi:10.1001/jama.2010.923 20628130

[17] Garwicz S, Anderson H, Olsen JH, ; Association of the Nordic Cancer Registries; Nordic Society for Pediatric Hematology Oncology. Late and very late mortality in 5-year survivors of childhood cancer: changing pattern over four decades—experience from the Nordic countries. Int J Cancer. 2012;131(7):1659-1666. doi:10.1002/ijc.27393 22170520

[18] Ludvigsson JF, Otterblad-Olausson P, Pettersson BU, Ekbom A. The Swedish personal identity number: possibilities and pitfalls in healthcare and medical research. Eur J Epidemiol. 2009;24(11):659-667. doi:10.1007/s10654-009-9350-y 19504049 PMC2773709

[19] Holmberg E, Holm LE, Lundell M, Mattsson A, Wallgren A, Karlsson P. Excess breast cancer risk and the role of parity, age at first childbirth and exposure to radiation in infancy. Br J Cancer. 2001;85(3):362-366. doi:10.1054/bjoc.2001.1868 11487266 PMC2364061

[20] Barlow L, Westergren K, Holmberg L, Talbäck M. The completeness of the Swedish Cancer Register: a sample survey for year 1998. Acta Oncol. 2009;48(1):27-33. doi:10.1080/02841860802247664 18767000

[21] Brooke HL, Talbäck M, Hörnblad J, . The Swedish cause of death register. Eur J Epidemiol. 2017;32(9):765-773. doi:10.1007/s10654-017-0316-1 28983736 PMC5662659

[22] Johansson LA. 2010. Dödsorsaksstatistik: historik, produktionsmetoder och tillförlitlighet. The National Board of Health and Welfare. Accessed March 12, 2022. https://www.socialstyrelsen.se/globalassets/sharepoint-dokument/artikelkatalog/statistik/2010-4-33.pdf

[23] Official Statistics of Sweden—Health and Diseases. Cancer incidence in Sweden 2011. Centre for Epidemiology. National Board of Health and Welfare. Accessed March 12, 2022. https://www.socialstyrelsen.se/globalassets/sharepoint-dokument/artikelkatalog/statistik/2012-12-19.pdf

[24] Ludvigsson JF, Andersson E, Ekbom A, . External review and validation of the Swedish national inpatient register. BMC Public Health. 2011;11:450. doi:10.1186/1471-2458-11-450 21658213 PMC3142234

[25] Altman DG, Bland JM. Interaction revisited: the difference between two estimates. BMJ. 2003;326(7382):219. doi:10.1136/bmj.326.7382.219 12543843 PMC1125071

[26] Pui CH, Campana D, Pei D, . Treating childhood acute lymphoblastic leukemia without cranial irradiation. N Engl J Med. 2009;360(26):2730-2741. doi:10.1056/NEJMoa0900386 19553647 PMC2754320

[27] Otterman G, Lahne K, Arkema EV, Lucas S, Janson S, Hellström-Westas L. Childhood death rates declined in Sweden from 2000 to 2014 but deaths from external causes were not always investigated. Acta Paediatr. 2019;108(1):160-168. doi:10.1111/apa.14309 29520820

[28] Oskarsson T, Söderhäll S, Arvidson J, ; Nordic Society of Paediatric Haematology and Oncology (NOPHO) ALL Relapse Working Group. Relapsed childhood acute lymphoblastic leukemia in the Nordic countries: prognostic factors, treatment and outcome. Haematologica. 2016;101(1):68-76. doi:10.3324/haematol.2015.131680 26494838 PMC4697893

[29] Bertuccio P, Alicandro G, Malvezzi M, . Childhood cancer mortality trends in Europe, 1990-2017, with focus on geographic differences. Cancer Epidemiol. 2020;67:101768. doi:10.1016/j.canep.2020.101768 32593162

[30] Wang H, Liddell CA, Coates MM, . Global, regional, and national levels of neonatal, infant, and under-5 mortality during 1990-2013: a systematic analysis for the Global Burden of Disease Study 2013. Lancet. 2014;384(9947):957-979. doi:10.1016/S0140-6736(14)60497-9 24797572 PMC4165626

[31] Hossain MJ, Xie L, McCahan SM. Characterization of pediatric acute lymphoblastic leukemia survival patterns by age at diagnosis. J Cancer Epidemiol. 2014;2014:865979. doi:10.1155/2014/865979 25309596 PMC4182848

[32] Williams LA, Spector LG. Survival differences between males and females diagnosed with childhood cancer. J Natl Cancer Inst Cancer Spectr. 2019;3(2):pkz032. doi:10.1093/jncics/pkz032 31259303 PMC6580869

[33] Gatta G, Botta L, Rossi S, ; EUROCARE Working Group. Childhood cancer survival in Europe 1999-2007: results of EUROCARE-5—a population-based study. Lancet Oncol. 2014;15(1):35-47. doi:10.1016/S1470-2045(13)70548-5 24314616

[34] Gustafsson G, Kreuger A. Sex and other prognostic factors in acute lymphoblastic leukemia in childhood. Am J Pediatr Hematol Oncol. 1983;5(3):243-250. 6578688

[35] Lanning M, Garwicz S, Hertz H, . Superior treatment results in females with high-risk acute lymphoblastic leukemia in childhood. Acta Paediatr. 1992;81(1):66-68. doi:10.1111/j.1651-2227.1992.tb12081.x 1600307

[36] Meeske KA, Ji L, Freyer DR, . Comparative toxicity by sex among children treated for acute lymphoblastic leukemia: a report from the Children’s Oncology Group. Pediatr Blood Cancer. 2015;62(12):2140-2149. doi:10.1002/pbc.25628 26173904 PMC4624005

[37] Wheeler K, Chessells JM, Bailey CC, Richards SM. Treatment related deaths during induction and in first remission in acute lymphoblastic leukaemia: MRC UKALL X. Arch Dis Child. 1996;74(2):101-107. doi:10.1136/adc.74.2.101 8660070 PMC1511507

[38] Oskarsson T, Söderhäll S, Arvidson J, ; Nordic Society of Paediatric Haematology and Oncology (NOPHO) ALL Relapse Working Group. Treatment-related mortality in relapsed childhood acute lymphoblastic leukemia. Pediatr Blood Cancer. 2018;65(4):e26909. doi:10.1002/pbc.26909 29230958

[39] Rostgaard K, Hjalgrim H, Madanat-Harjuoja L, Johannesen TB, Collin S, Hjalgrim LL. Survival after cancer in children, adolescents and young adults in the Nordic countries from 1980 to 2013. Br J Cancer. 2019;121(12):1079-1084. doi:10.1038/s41416-019-0632-1 31719686 PMC6964683

