# Supplementary material for: Mortality Among Pediatric Patients With Acute Lymphoblastic Leukemia in Sweden From 1988 to 2017
Source: JAMA Netw Open. 2022 Nov 28;5(11):e2243857. doi: 10.1001/jamanetworkopen.2022.43857 (PMC9706364; doi:10.1001/jamanetworkopen.2022.43857)
Supplement: Supplement. — eTable 1. Inclusion Criteria Used in the Cancer Register and the Patient Register eTable 2. Codes for Cause-specific Mortality [file jamanetwopen-e2243857-s001.pdf]

Supplementary Online Content

Björk-Eriksson T, Boström M, Bryngelsson IL, et al. Mortality among pediatric patients with acute lymphoblastic leukemia in Sweden from 1988 to 2017. *JAMA Netw Open*. 2022;5(11):e2243857. doi:10.1001/jamanetworkopen.2022.43857

eTable 1. Inclusion Criteria Used in the Cancer Register and the Patient Register

eTable 2. Codes for Cause-specific Mortality

This supplementary material has been provided by the authors to give readers additional information about their work.

© 2022 Björk-Eriksson T et al. *JAMA Network Open*.

eTable 1. Inclusion Criteria Used in the Cancer Register and the Patient Register

**Registers**	**Codes**
Cancer Register	
ICD-7 codes	204.0, 204.1, 204.9, 207.0, 207.9
PAD codes	206, 296, 826, 996
Patient Register	
ICD-10 codes	C91.0, C91.1, C91.3, C91.5, C91.7, C91.9, C95.0, C95.9
ICD-9 codes	204.0, 204.1, 204.9, 208.0, 208.9

Abbreviations: ICD, International Classification of Diseases; PAD, Pathological-Anatomical Diagnosis.

© 2022 Björk-Eriksson T et al. *JAMA Network Open*.

eTable 2. Codes for Cause-specific Mortality

Cause-specific mortality	ICD-10	ICD-9
Malignant tumors	C00-C97	140-208
Malignant neoplasm of the brain	C71	191
Malignant neoplasm of lymphoid, hematopoietic, and related tissues	C90-C96	204-208
Ischemic heart disease	I20-I25	410-414
Cerebrovascular disease	I61-I64	431-434, 436

Abbreviation: ICD, International Classification of Diseases.

© 2022 Björk-Eriksson T et al. *JAMA Network Open*.
